# Identification of an immune checkpoint gene signature that accurately predicts prognosis and immunotherapy response in endometrial carcinoma

**DOI:** 10.18632/aging.203189

**Published:** 2021-06-22

**Authors:** Shaowen Li, Chunli Dong, Jiayan Chen, Xiaocui Gao, Xiuying Xie, Xin Zhang

**Affiliations:** 1 Department of Pediatrics, the Second Affiliated Hospital of Xi’an Jiaotong University, Xi’an, China; 2 Department of Obstetrics and Gynecology, the Second Affiliated Hospital of Xi’an Jiaotong University, Xi’an, China

**Keywords:** endometrial carcinoma, prognosis, immunotherapy, immune checkpoint genes, biomarkers

## Abstract

In this study, we performed a bioinformatics analysis to identify immune checkpoint genes (ICGs) associated with prognosis and the immunotherapeutic response in endometrial carcinoma (EC) patients. We classified 47 ICGs into high, medium, and low expression groups by performing RNA-sequencing data analysis of EC patient samples from The Cancer Genome Atlas (*n* = 521) and GSE77688 (*n* = 88) datasets. Univariate Cox regression analysis showed that seven ICGs (*VTCN1, TNFRSF18, TNFRSF14, TNFRSF4, CD40LG, TMIGD2,* and *BTLA*) were associated with prognosis in EC patients. Spearman correlation analysis showed that prognosis-related ICGs correlated positively with immunotherapy response factors, including tumor mutation burden (TMB), mismatch repair gene mutations, neoantigens, clinical stages, and adaptive immune resistance pathway genes. We identified a prognostic gene signature of four ICGs (*IDO1, CD274, CTLA4,* and *TNFRSF14*) that accurately predicted survival outcomes of EC patients. TIMER database and Kaplan-Meier survival analysis showed that OS among EC patients with low *TNFRSF14* expression was significantly shorter than among those with high *TNFRSF14* expression. *In vitro* experiments showed that *TNFRSF14* silencing increased the migration and invasiveness of EC cells by promoting epithelial-mesenchymal transition (EMT). Collectively, these findings reveal an immune checkpoint gene signature that accurately predicts survival outcomes and immunotherapeutic responses in EC patients.

## INTRODUCTION

Endometrial carcinoma (EC) is a common cancer of the female reproductive system and ranks sixth among most commonly diagnosed cancers in females, with constantly increasing rates of incidence worldwide [1]. The 5-year survival rate of patients with early-stage EC that undergo curative surgery is 74–91% [2]. However, despite undergoing chemotherapy, radiotherapy, and other treatments, the 5-year survival rate of patients with metastasized or relapsed EC is only 20–26% [3]. Therefore, new and more effective treatments are urgently required for EC patients. In recent years, immunotherapy has emerged as an effective treatment strategy for several solid cancers [4]. For example, monoclonal antibodies against immune checkpoint proteins such as PD-1 and PD-L1 have emerged as frontline treatments of several types of solid tumors. In EC however, clinical trials are still underway to determine safety and efficacy of most immune checkpoint inhibitor therapies.

Aberrant expression of immune checkpoint proteins is a well-known tumor immune escape mechanism [5]. Inhibitory ligands expressed on the surface of tumor cells, dendritic cells, macrophages, and bone marrow-inhibiting cells bind to the surface receptor molecules on the T cells and activate signaling pathways that inhibit proliferation and anti-tumor activity of T cells [6]. Previous studies have shown that immune checkpoint pathways such as PD-1/PD-L1, CTLA-4/CD80, TIM-3/Gal-9, and LAG-3/FGL1 are associated with tumor immune escape mechanisms [7–9]. Current studies on immune checkpoint therapy in EC are focused on targeting the PD-1/PD-L1 pathway.

Programmed cell death protein 1 (PD-1) is an important immunosuppressive receptor protein that is mainly expressed on the surface of T cells, B cells, and macrophages; programmed death-ligand 1 (PD-L1) is the binding partner of PD-1 and is highly expressed on the surface of tumor cells [10, 11]. The use of monoclonal anti-PD-1 antibody (pembrolizumab) is recommended as an alternative treatment option by the National Comprehensive Cancer Network (NCCN) clinical practice guidelines in oncology for patients with MSI-H/dMMR endometrial cancer.

Currently, the overall effectiveness of immunotherapy in EC remains limited [12, 13]. Therefore, analysis of the expression of various immune checkpoint molecules is necessary to identify patients that may respond to specific immunotherapies. The Cancer Genome Atlas (TCGA) data in the United States shows that EC patients can be classified into four main subtypes: (1) *POLE* hypermutation; (2) high rate of mutations and microsatellite instability (MSI); (3) high copy number mutations in genes such as *TP53*; and (4) low copy number mutations. EC patients with high tumor mutation burden (TMB) are more likely to benefit from treatment with PD-1/PD-L1 inhibitors [14–16]. Moreover, levels of tumor-infiltrated T cells, CD8^+^ T cells, and PD-1 are significantly higher in *POLE*-mutated EC tissues [17]. Furthermore, therapeutic efficacy of the PD-1 monoclonal antibody is significantly higher in *POLE*-mutant EC subtypes [18, 19]. This suggests that *POLE*-mutated and MSI subtypes are best suited for PD-1/PD-L1 inhibitor therapy. However, expression levels of various immune checkpoint genes (ICGs) in EC tissues are not well studied.

In this study, we analyzed the relationship between expression levels of 47 ICGs, prognosis, and clinicopathological parameters including immunotherapy response biomarkers such as mismatch repair (MMR) gene mutations and TMB.

## RESULTS

### Identification of high, medium, and low expressing ICGs in EC samples from the TCGA database

**Figure 1:**
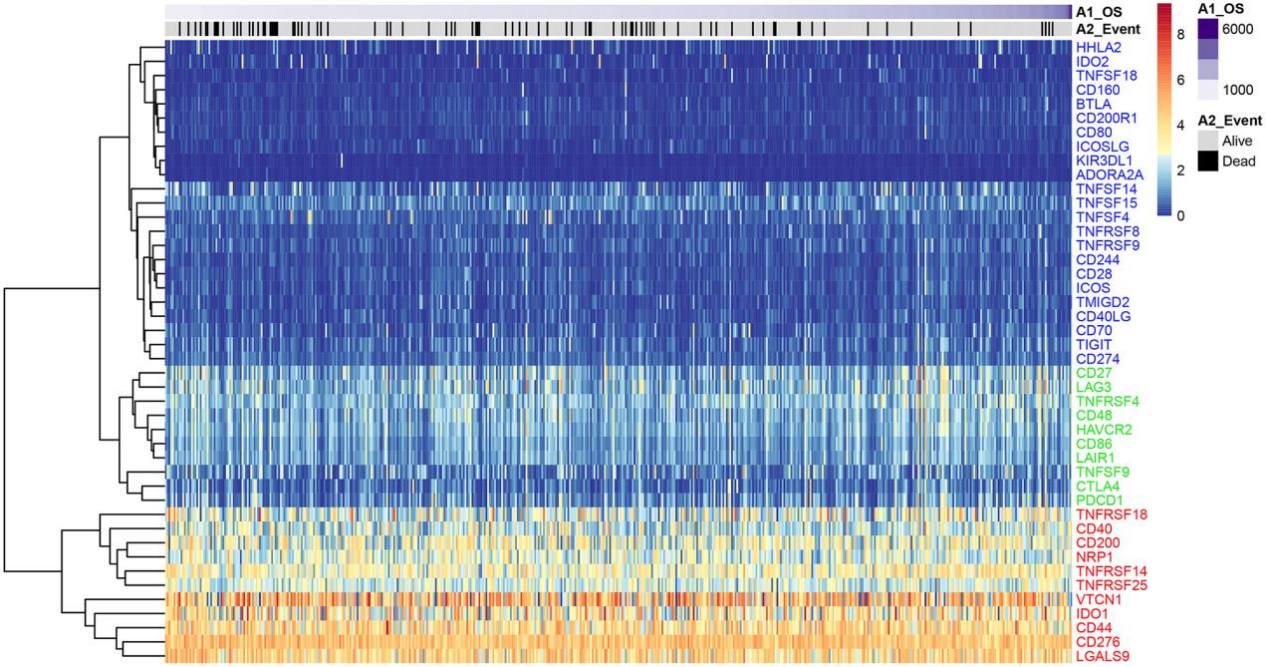
**ICG expression in the EC samples from the TCGA dataset.** Heat map shows expression levels of 47 ICGs in the TCGA-EC dataset (*n* = 521). Red: high expression group; green: medium expression group; blue: low expression group. The abscissa (x-axis) represents number of samples and ICGs are listed along the ordinate (y-axis).

We analyzed expression levels of 47 ICGs in EC samples from the TCGA database (*n* = 521) and identified high (red), medium (green), and low (blue) expression groups (Figure 1). The high expression ICGs included *VTCN1*, *IDO1*, and *CD44*, which were highly expressed in all EC tissue samples. The medium expression ICGs included *CD27* and *LAG3* whose expression varied significantly among EC tissue samples. The low expression ICGs included *KIR3DL1* and *ADORA2A* whose expression was significantly lower in most EC samples.

### Identification of seven prognosis-associated ICGs in EC

**Figure 2:**
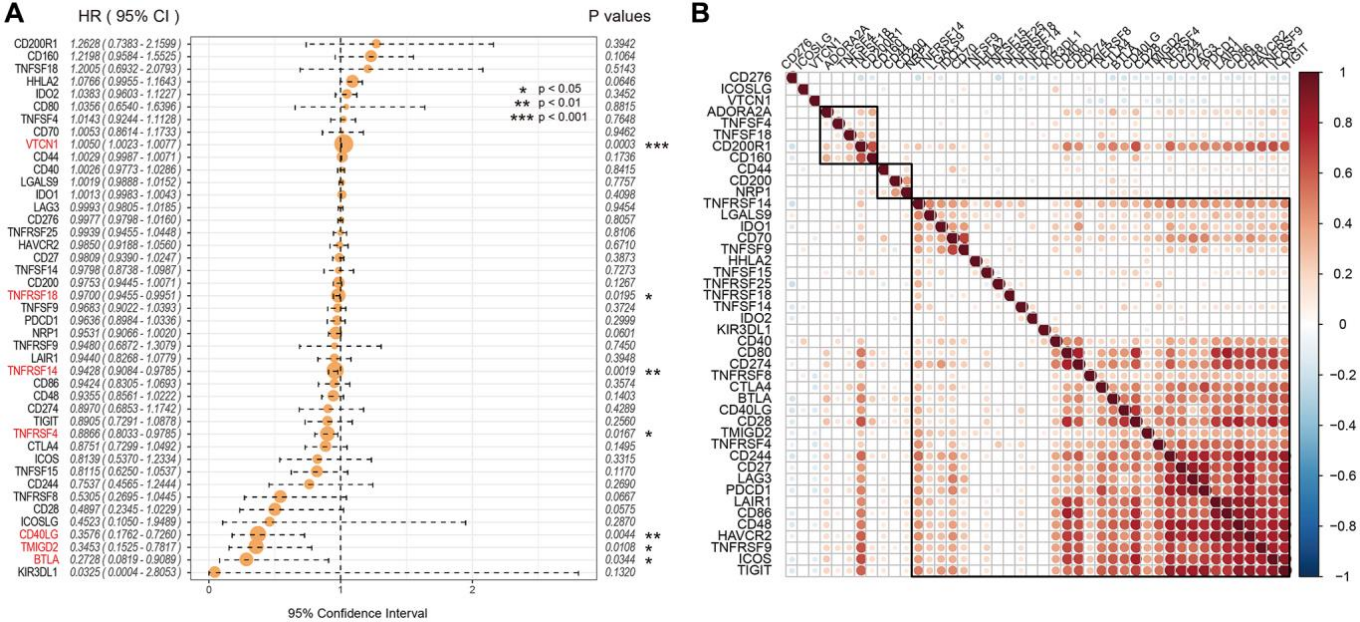
**Association between ICGs and prognosis of EC patients from the TCGA dataset.** (**A**) Univariate Cox regression analysis shows association between ICGs and prognosis of EC patients. (**B**) Spearman correlation analysis shows the relationship between various ICG pairs in EC tissues from the TCGA dataset. Note: Only ICG gene pairs with significant correlations are displayed; blank indicates insignificant correlation.

Univariate Cox regression analysis showed that seven out of 47 ICGs were significantly associated with prognosis of UCEC patients (log-rank *p* < 0.05; Figure 2A). Spearman correlation analysis showed that expression levels of the 7 ICGs were positively associated with prognosis of EC patients; ICGs also demonstrated additive effects, thereby suggesting synergistic co-operation between them (Figure 2B).

### The expression patterns of ICGs in the GEO-EC dataset was identical to the TCGA-EC dataset

**Figure 3:**
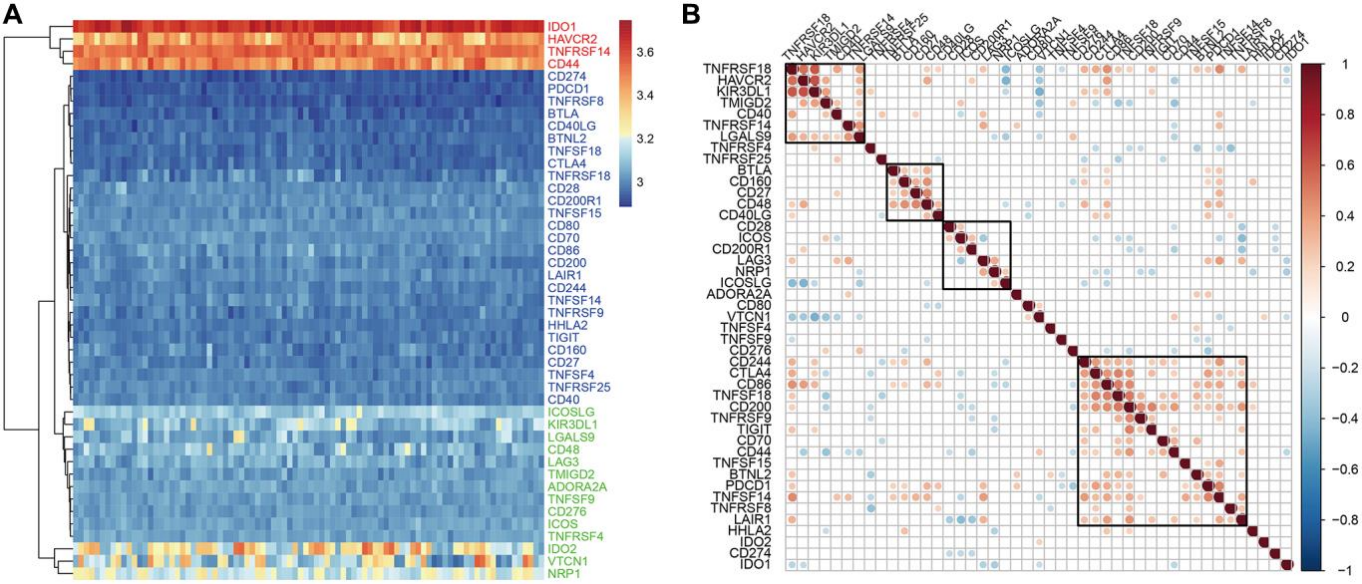
**ICG expression in EC samples from the GSE77688 dataset.** (**A**) Heat map shows expression levels of 47 ICGs in EC tissues from the GSE77688 data set. Red: high expression group; green: medium expression group; blue: low expression group. (**B**) Spearman correlation analysis shows relationship between various ICG pairs in EC tissues from the GSE77688 dataset. Note: Only ICG gene pairs with significant correlations are displayed; blank represents insignificant correlation.

The analysis of the expression levels of 47 ICGs in the GSE77688 dataset of EC samples also showed high, medium, and low expression ICG groups, which was similar to those identified in the TCGA dataset (Figure 3A). In both datasets, *TNFRSF14*, *IDO1*, and *CD44* were highly expressed; *LAG3*, *TNFRSF4*, *CD48*, and *TNFSF9* were moderately expressed; and *TNFSF18*, *HHLA2*, *BTLA*, and *CD160* showed low expression in EC samples (Figure 3A). Moreover, expression levels of the seven prognostic ICGs correlated with prognosis of EC patients in the GSE77688 data set; moreover, these prognosis-related ICGs showed synergistic effects (Figure 3B).

### Prognostic ICGs are positively correlated with TMB in EC patients

**Figure 4:**
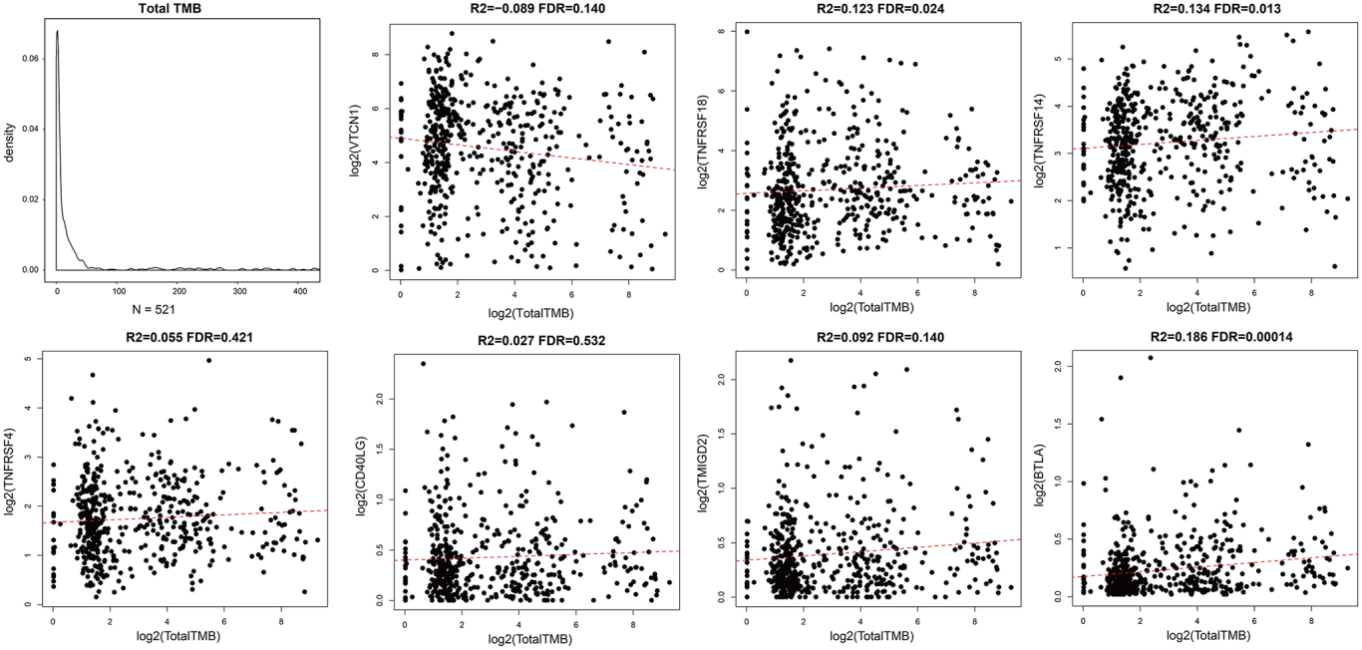
**Association between prognostic ICGs and TMB in EC.** Scatter plot shows relationship between expression levels of seven prognostic ICGs and TMB. R2, correlation coefficient; FDR, false detection rate. The abscissa represents log_2_ (Total TMB), and ordinate represents expression levels of seven prognostic ICGs in EC tissues from the TCGA dataset.

We calculated tumor mutation burden (TMB) of EC samples using TCGA somatic mutation data after excluding intron intervals and silent mutations. We then performed Spearman correlation analysis to determine the association between TMB and prognosis-associated ICGs. The distribution of TMB was non-normal (Shapiro test: *p* value <1e-5). TMB and ICG expression data of EC samples is shown in Supplementary Table 1. Overall, we observed positive correlation between TMB and most of the prognosis-associated ICGs, especially *TNFRSF18*, *TNFRSF14*, and *BTLA* (R2 >0, FDR <0.05; Figure 4). TMB was most significantly and positively correlated with *BTLA* (FDR <0.0005; Figure 4). Among the seven prognostic ICGs, only *VTCN1* showed negative correlation with TMB (Figure 4).

### Expression levels of ICGs in EC tissues correlate with mutations in MMR genes

**Figure 5:**
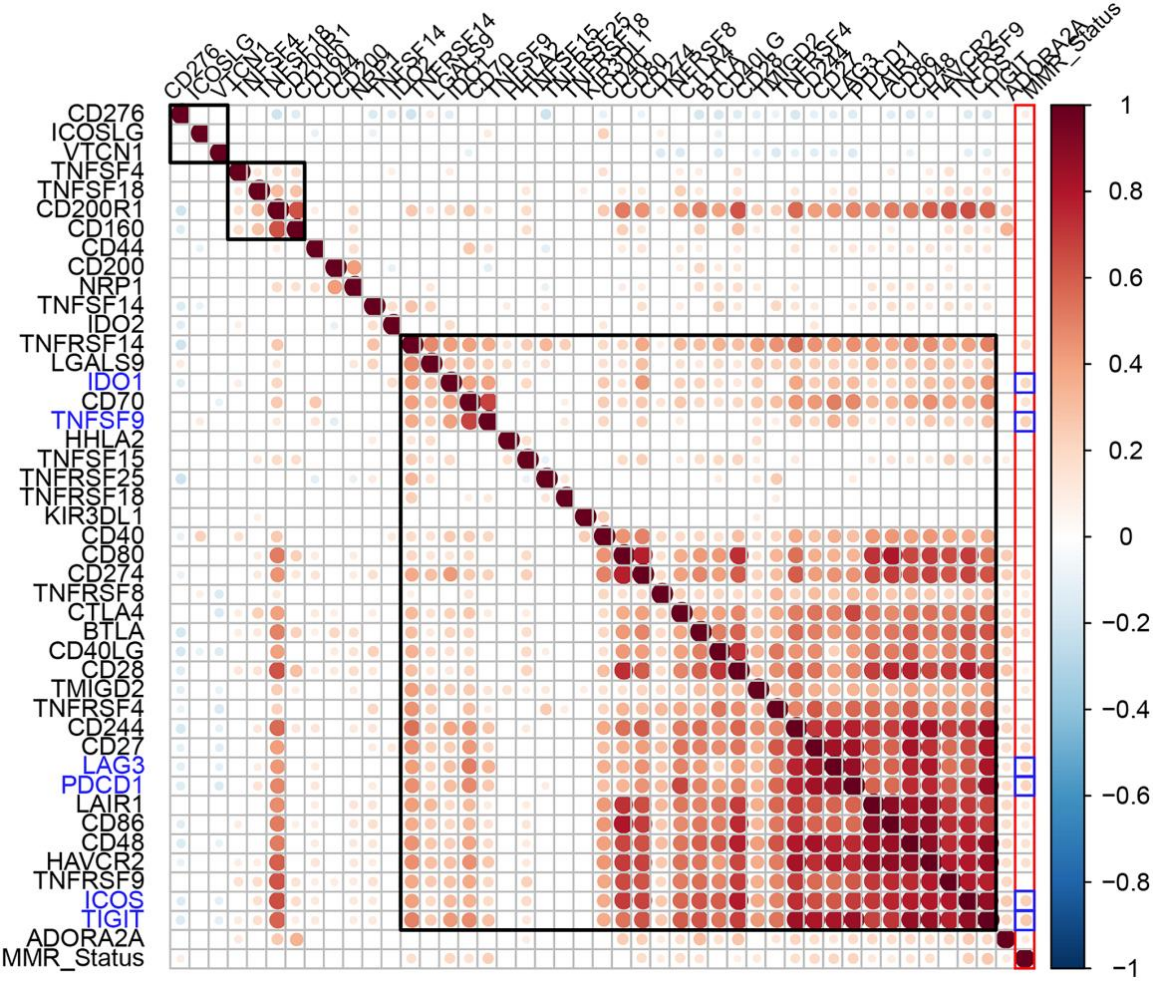
**Association between ICGs and MMR gene mutations in EC.** Spearman correlation analyses between expression of ICGs and MMR gene mutations in EC tissues. Larger dots indicate stronger correlations. Red indicates positive correlation and blue indicates negative correlation. The color intensity indicates strength of positive or negative correlations.

Aberrant function of mismatch repair (MMR) genes increases somatic mutations in cells and is associated with tumorigenesis. We analyzed somatic mutation data of EC samples from the TCGA database to determine the relationship between the expression levels of ICGs and mutations in five MMR genes (*MLH1*, *MSH2*, *MSH6*, *PMS2*, *and EPCAM*). We identified MMR gene mutations in EC samples (Supplementary Table 2). We observed positive correlation between mutations in MMR genes and expression levels of ICGs, especially *IDO1*, *TNFSF9*, *LAG3*, *PDCD1*, *ICOS*, and *TIGIT* (R2 >0.2; Figure 5). Among these, *IDO1* and *PDCD1* are closely related to immune regulation.

### Expression levels of ICGs in EC tissues correlate with levels of neoantigens

**Figure 6:**
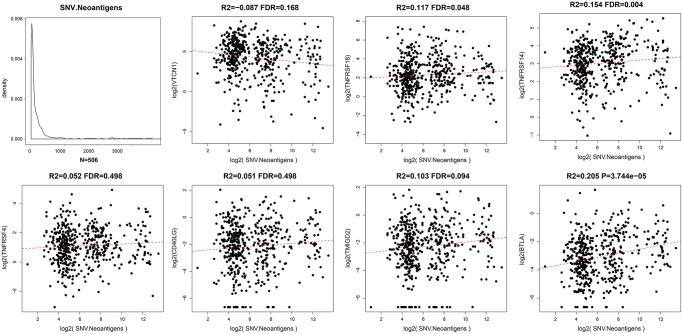
**Relationship between ICGs and neoantigens in EC.** Scatter diagram demonstrates correlation between expression levels of ICGs and neoantigens in EC tissues. R2 refers to the correlation coefficient; FDR indicates false detection rate. The abscissa represents log_2_ (expression of neoantigens) and ordinate represents expression levels of different ICGs.

Somatic mutations in protein-encoding genes of tumor cells causes synthesis of proteins with altered sequences. The peptides generated from mutated proteins are called neoantigens. These neoantigens are presented on the surface of tumor cells by the major histocompatibility class I complex (MHC I) and are recognized as foreign by T cells through the T-cell receptors. Hence, they trigger tumor-specific adaptive immune responses. We used the TCGA somatic mutation data to further analyze the relationship between neoantigens and expression levels of ICGs in EC tissues (Supplementary Table 3). We observed positive correlation between neoantigens and ICGs such as *TNFRSF18*, *TNFRSF14*, and *BTLA* (R2 >0, FDR <0.05; Figure 6). These results were consistent with previous results that showed positive correlation between TMB and expression of ICGs such as *TNFRSF18*, *TNFRSF14*, and *BTLA*.

### The expression of ICGs in EC tissues is associated with adaptive immune resistance pathway genes

**Figure 7:**
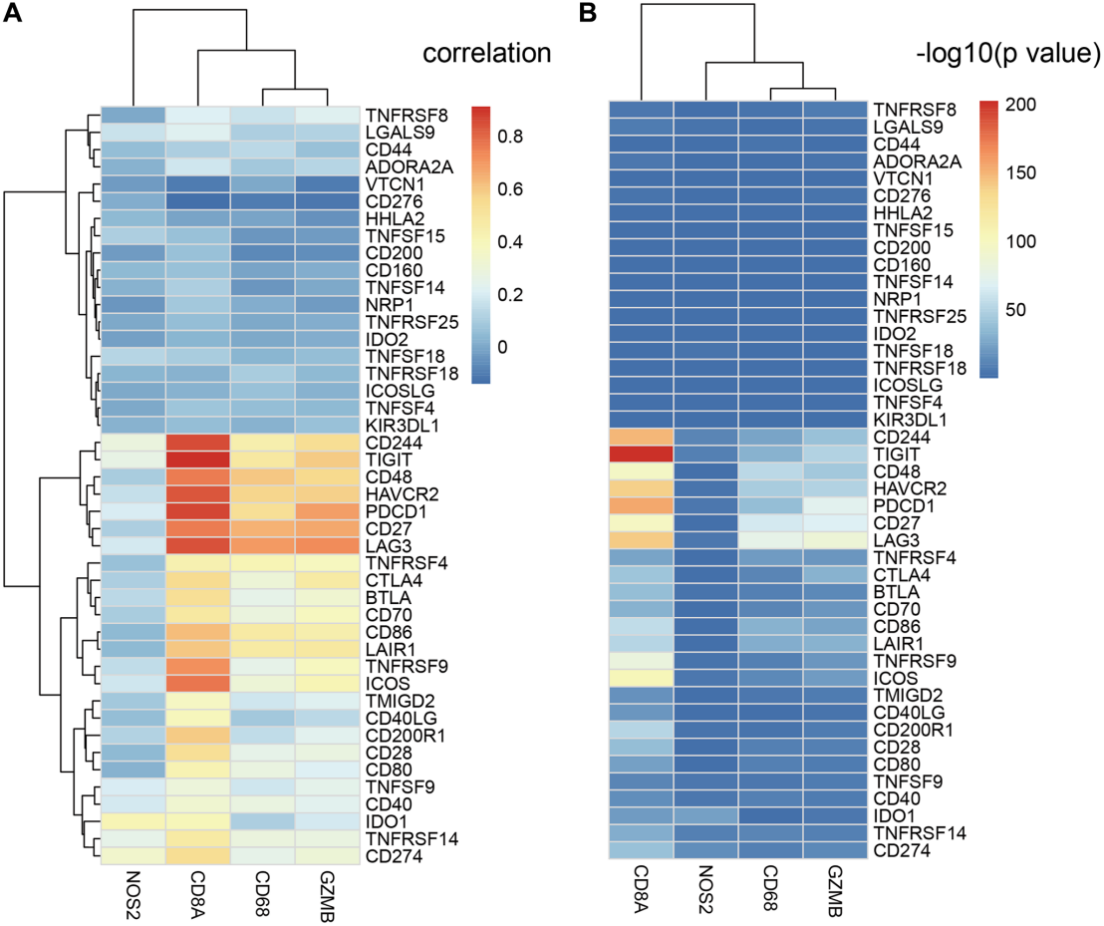
**Relationship between ICGs and adaptive immune resistance pathway genes in EC.** (**A**) Heat map shows correlation coefficients of ICGs and adaptive immune resistance pathway genes. (**B**) *P*-values for the correlation coefficients show the significance of the association between ICGs and adaptive immune resistance pathway genes. The -log_10_ *P*-values are plotted along the x-axes.

CD8^+^ T cells produce interferon-γ, which upregulates expression of adaptive immune resistance pathway genes such as PD-1/PD-L1 axis and *IDO1*. We analyzed the correlation between ICGs and adaptive immune resistance pathway genes such as *CD8A*, *GZMB*, *CD68*, and *NOS2*. We observed positive correlation between expression levels of adaptive immune resistance pathway genes (*CD8A*, *CD244*, *TIGIT*, and *PDCD1*) and expression levels of ICGs in EC tissues (Figure 7A). The correlations between these three adaptive immune resistance pathway genes (*CD8A*, *TIGIT*, and CD244) and expression levels of ICGs were statistically significant (*p* <1e-5; Figure 7B). The details of the correlation analysis are shown in Supplementary Table 4.

### The expression levels of prognostic ICGs are associated with clinicopathological characteristics of EC patients

**Figure 8:**
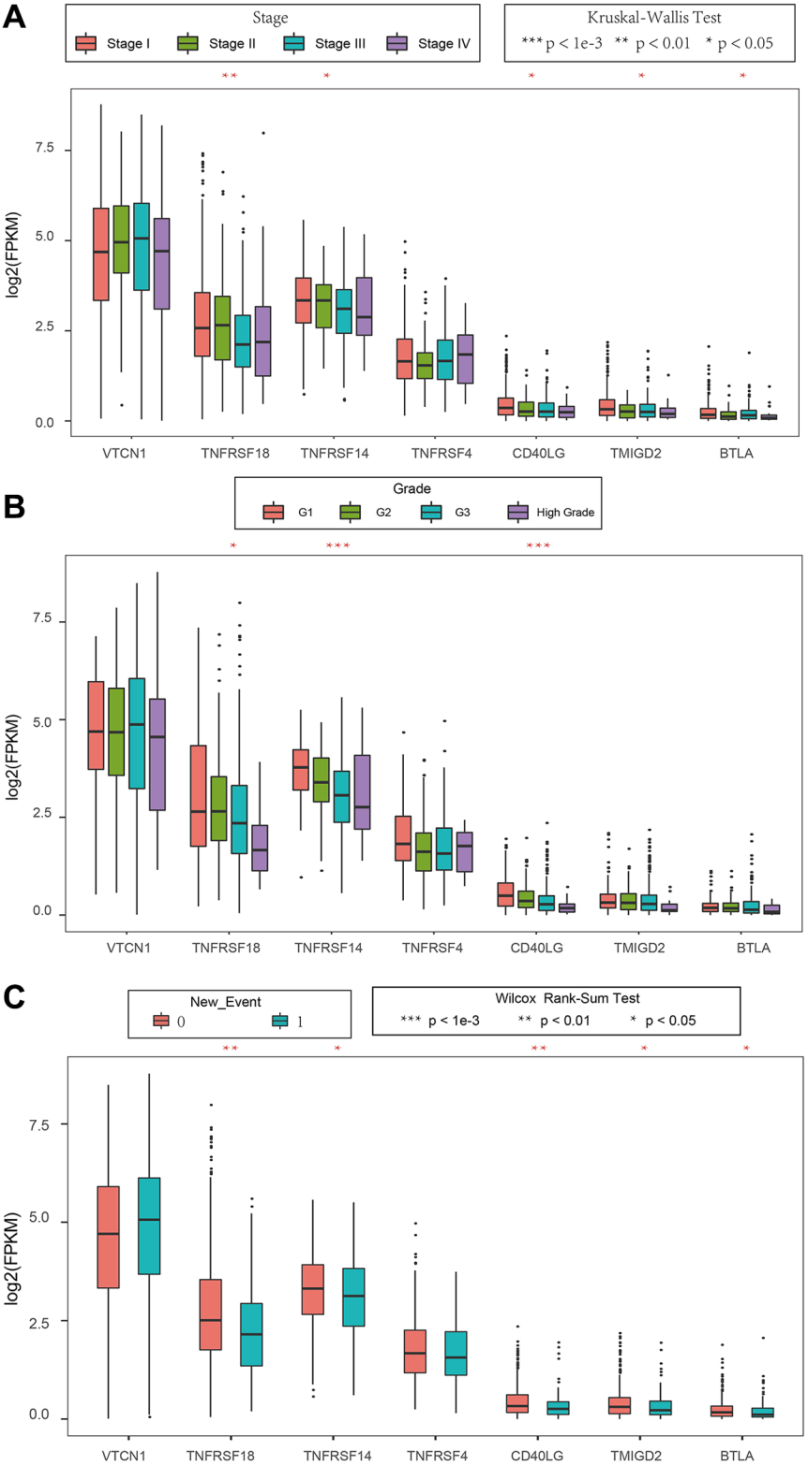
**Association between ICGs and clinicopathological variables in EC.** (**A**–**C**) Box plot shows expression values (FPKM) of seven prognostic ICGs in various (**A**) tumor stages, (**B**) tumor grades, and (**C**) new events of EC patients. The abscissa represents ICGs and ordinate represents their gene expression values.

We then analyzed the relationship between prognostic ICGs and clinicopathological characteristics of EC patients using the clinical data from the TCGA database. We focused on expression levels of these seven prognostic ICGs in different tumor stages, tumor grades, and new events. The seven prognostic ICGs included 4 medium-expressing ICGs and 3 low-expressing ICGs (Supplementary Figure 1). The expression levels of *TNFRSF18*, *TNFRSF14*, and *CD40LG* showed significant differences based on clinical characteristics such as tumor stages, tumor grades, and new events Figure 8A–8C (*p* < 0.05).

### Identification of EC subtypes based on the expression of ICGs

Our previous results showed that *TNFRSF14* was significantly associated with prognosis of TCGA-EC samples, TMB, neoantigens, and clinicopathological characteristics of EC patients. *TNFRSF14* also showed positive correlation with *CD8A*. This suggested that *TNFRSF14* may regulate the expression of adaptive immune resistance pathway genes. Therefore, we analyzed the relationship between prognosis of EC patients and the combinatorial expression status of *TNFRSF14*, *CD274*, *CTLA4,* and *IDO1*.

**Figure 9:**
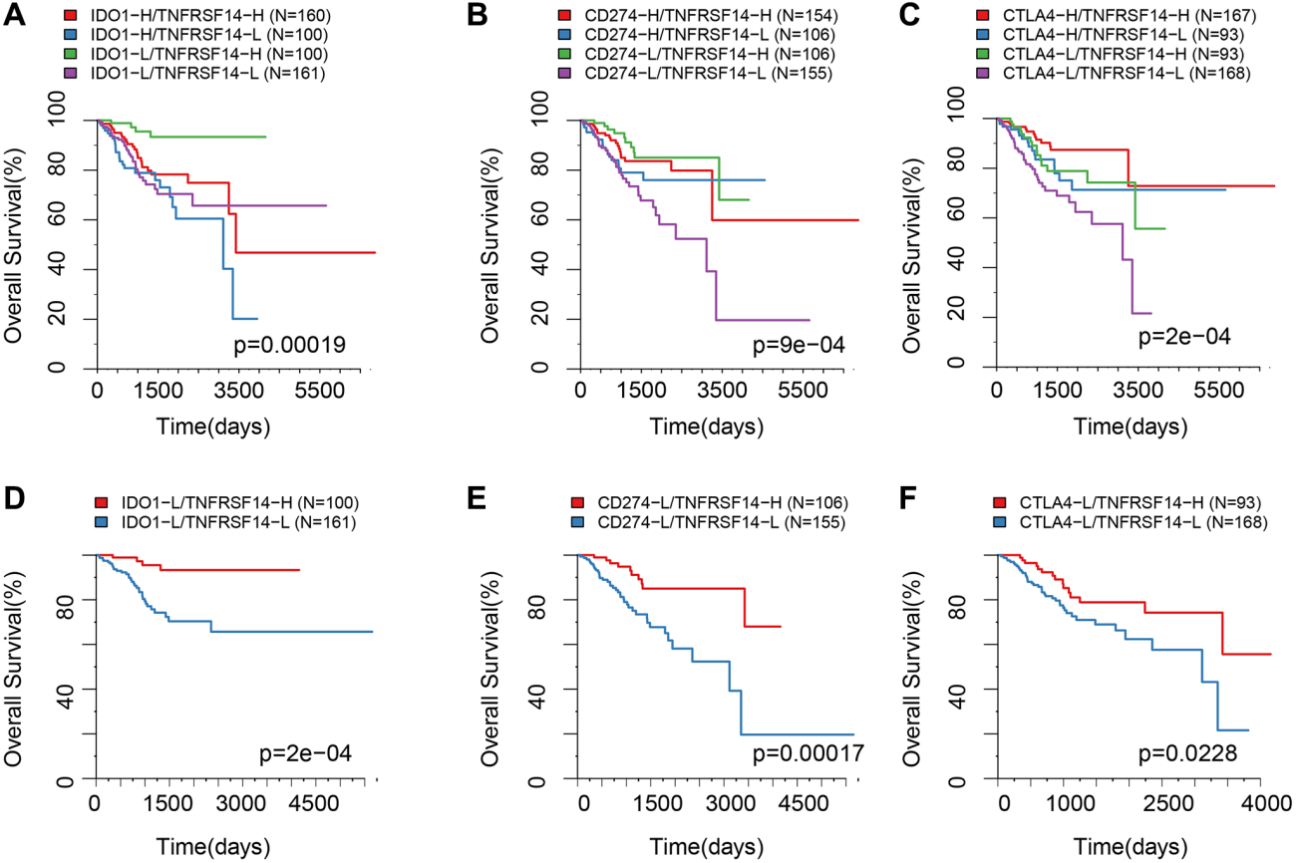
**Survival analysis of EC patients belonging to different ICG expression subgroups.** (**A**) Kaplan-Meier survival curves show OS of EC patients belonging to high or low *IDO1* and *TNFRSF14* expression groups. (**B**) Kaplan-Meier survival curves show OS of EC patients belonging to high or low *CD274* and *TNFRSF14* expression groups. (**C**) Kaplan-Meier survival curves show OS of EC patients belonging to high or low *CTLA4* and *TNFRSF14* expression groups. (**D**) Kaplan-Meier survival curves show OS of EC patients belonging to *IDO1*^low^ *TNFRSF14*^high^ and *IDO1*^low^ *TNFRSF14*^low^ expression groups. (**E**) Kaplan-Meier survival curves show OS of EC patients belonging to *CD274*^low^ *TNFRSF14*^high^ and *CD274*^low^ *TNFRSF14*^low^ expression groups. (**F**) Kaplan-Meier survival curves show OS of EC patients belonging to *CTLA4*^low^ *TNFRSF14*^high^ and *CTLA4*^low^ *TNFRSF14*^low^ expression groups. The abscissa represents survival time, and the ordinate represents overall survival.

EC patients with high *TNFRSF14* expression and low *IDO1*, *CD274*, and *CTLA4* expression showed better prognosis, whereas, EC patients with low *TNFRSF14*, *IDO1*, *CD274*, and *CTLA4* expression showed worse prognosis (Figure 9A–9C). Furthermore, we observed significant differences in the OS rates of EC patients belonging to these two groups (Figure 9D–9F).

### TNFRSF14 expression significantly correlates with prognosis and clinicopathological characteristics of EC patients

**Figure 10:**
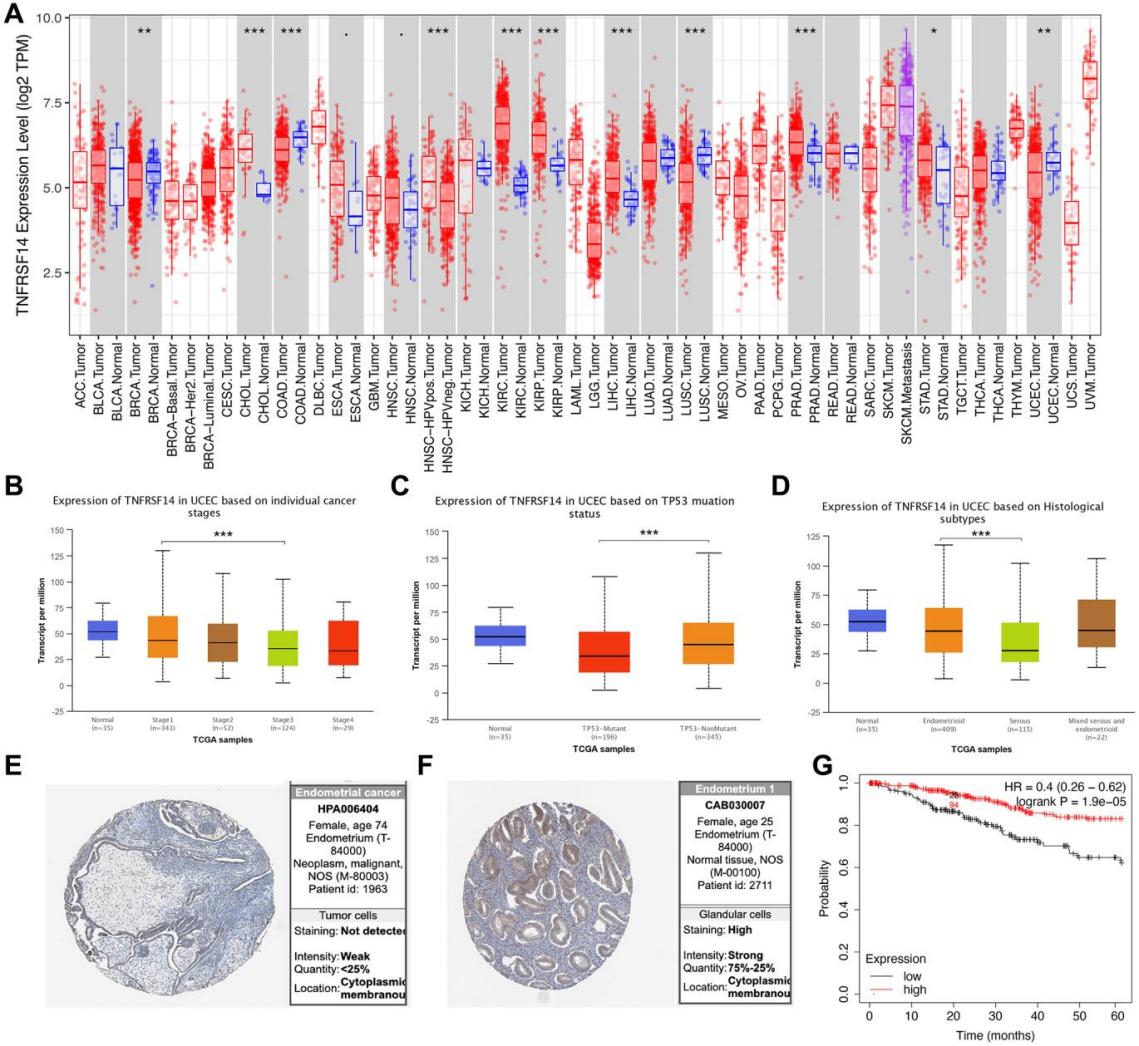
**Relationship between *TNFRSF14* expression and prognosis of EC patients.** (**A**) *TNFRSF14* expression in pan-carcinoma tissues. (**B**) Relationship between *TNFRSF14* expression and clinical stages of endometrial cancer. (**C**) Relationship between *TNFRSF14* expression and *TP53* mutations in EC. (**D**) Relationship between *TNFRSF14* expression and tissue subtypes of EC. (**E**) *TNFRSF14* expression in endometrial cancer tissues. (**F**) *TNFRSF14* expression in normal endometrial tissues. (**G**) Kaplan-Meier survival analysis shows the relationship between *TNFRSF14* expression and prognosis of EC patients using the KM Plotter database.

TIMER database analysis showed that expression of TNFRSF14 was significantly downregulated in breast cancer, colon adenocarcinoma, lung squamous cell carcinoma, and EC tissues, and significantly upregulated in cholangiocarcinoma, kidney renal clear cell carcinoma, kidney renal papillary cell carcinoma, liver hepatocellular carcinoma, and prostate adenocarcinoma tissues compared to their corresponding normal tissues (Figure 10A). *TNFRSF14* expression was significantly higher in early-stage EC patients compared to advanced-stage EC patients. *TNFRSF14* expression was significantly higher in EC tissues with wild-type *TNFRSF14* compared to those with mutant *TNFRSF14;* moreover, *TNFRSF14* expression was significantly lower in serous tissues compared to the endometrioid tissues (Figure 10B–10D). TNFRSF14 protein expression was also significantly lower in EC tissues compared to normal endometrial tissues (Figure 10E–10F). Kaplan-Meier survival curve analysis showed that overall survival of EC patients with low TNFRSF14 expression was significantly shorter than those with high TNFRSF14 expression (Figure 10G).

### TNFRSF14 silencing promotes EC cell progression by enhancing EMT

**Figure 11:**
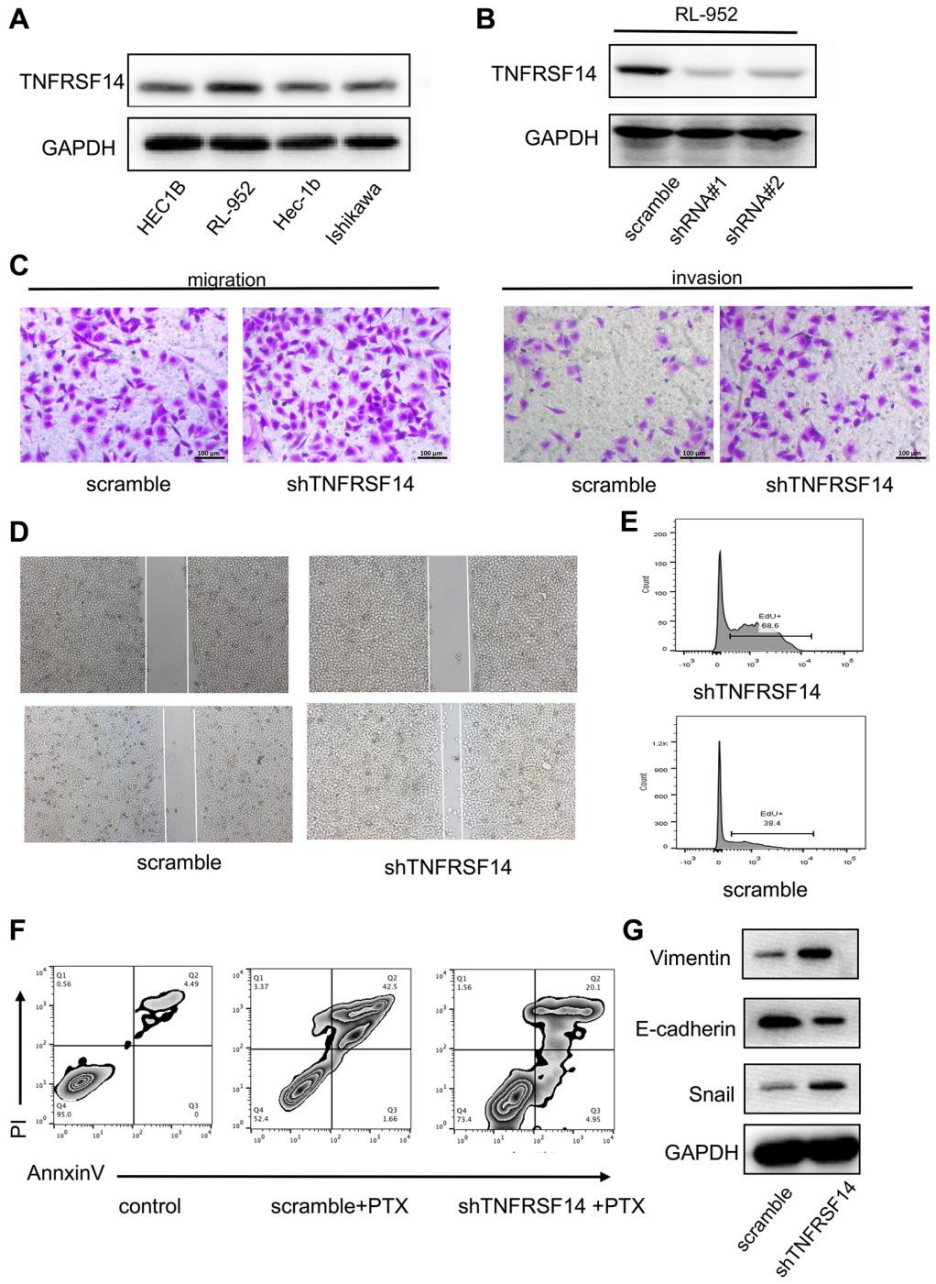
***TNFRSF14* knockdown promotes EC progression by enhancing EMT.** (**A**) Western blot analysis shows TNFRSF14 protein expression in EC cell lines (HEC1B, RL-952, Hec-1B, and Ishikawa). (**B**) Western blot analysis shows TNFRSF14 protein levels in control and *TNFRSF14-*shRNA#1- and *TNFRSF14-*shRNA#2-transfected RL-952 cells. (**C**–**D**) Transwell invasion assay and wound healing assay results show the invasiveness and migration ability of control and *TNFRSF14-*silenced RL-952 cells. (**E**) EdU incorporation assay results show the proliferation rates of control and *TNFRSF14-*silenced RL-952 cells. (**F**) Flow cytometry analysis shows the percentage apoptosis of 5-fluorouracil-treated control and *TNFRSF14* knockdown RL-952 cells. (**G**) Western blot analysis shows expression levels of EMT-associated marker proteins, Vimentin and Snail, in control and *TNFRSF14-*silenced RL-952 cells.

We then performed *in vitro* experiments to confirm results from the bioinformatics analysis. We first evaluated expression of TNFRSF14 in four EC cell lines, namely, HEC1B, RL-952, Hec-1B, and Ishikawa. Western blot analysis showed that TNFRSF14 protein levels were significantly higher in the RL-952 cell line compared to the other EC cell lines (Figure 11A). Therefore, we used RL-952 cell line for subsequent experiments.

We investigated the biological function of TNFRSF14 by using shRNAs targeting *TNFRSF14*. Western blot analysis showed that *TNFRSF14* knockdown effect was significantly higher with shRNA#1 compared to shRNA#2 (Figure 11B). Therefore, we used shRNA #1 for subsequent experiments. *In vitro* Transwell migration and invasion assays showed that *TNFRSF14* silencing increased migration and invasion capacity of EC cells (Figure 11C). Wound healing assay confirmed that *TNFRSF14* silencing significantly increased migration ability of EC cells (Figure 11D). Moreover, 5-ethynyl-2’-deoxyuridine (EdU) incorporation assay results showed that *TNFRSF14* knockdown significantly increased proliferation of EC cells (Figure 11E). Paclitaxel is currently used as a chemotherapeutic agent in EC treatment. Therefore, we studied the effects of *TNFRSF14* knockdown on paclitaxel resistance of EC cells. Knockdown of *TNFRSF14* decreased apoptosis rate of paclitaxel-treated EC cells (Figure 11F). This suggested that *TNFRSF14* downregulation increased chemoresistance of EC cells to 5-fluorouracil.

We then investigated underlying mechanisms by which *TNFRSF14* regulates EC progression. Epithelial-mesenchymal transition (EMT) is a key event associated with cancer progression and metastasis. Western blot analysis showed that EMT-associated markers such as Vimentin and Snail were upregulated in *TNFRSF14*-silenced EC cells (Figure 11G). This suggested that *TNFRSF14* promoted EC cell progression by enhancing EMT.

## DISCUSSION

EC is a common malignancy in women, with increasing incidence rates in the last few decades. Comprehensive treatment strategies including surgery have significantly improved prognosis of patients with early-stage EC [20]. However, prognosis of patients with advanced-stage EC and high-risk factors remains poor because of increased metastasis and recurrence despite availability of several treatment options such as chemotherapy, radiotherapy, and hormone therapy [21]. In recent years, clinical trials with drugs targeting immune checkpoint proteins such as PD-1 and PD-L1 have shown greater efficacy and tolerance in EC patients [22, 23]. However, studies have also shown that many cancer patients do not benefit from immunotherapy. Many studies have shown that expression levels of PD-1 and PD-L1 alone cannot accurately identify patients that may benefit from immunotherapy [24]. Therefore, comprehensive analysis of immune checkpoint proteins in EC tissues is required to identify more accurate predictive markers of immunotherapeutic response.

Immune checkpoint proteins are membrane proteins that play a key role in immune homeostasis by regulating activation of immune cells [25]. Effector T cells modulate expression of several immunosuppressive proteins to prevent hyper-activation because it can cause autoimmunity and other immune-related disorders [26]. Drugs targeting immune checkpoint proteins have demonstrated significantly higher response in clinical trials of several cancers [25–27].

In this study, we comprehensively analyzed expression of 47 ICGs in EC patient tissues from the TCGA and GEO databases. Based on the results, we classified ICGs into high, medium, and low expression groups. We identified seven ICGs, namely, *VTCN1*, *TNFRSF18*, *TNFRSF14*, *TNFRSF4*, *CD40LG*, *TMIGD2*, and *BTLA*, which showed association with prognosis of EC patients. We also observed synergistic relationship between the ICGs.

Immunotherapy is emerging as an effective therapeutic option for several cancers. Hence, several studies have focused in identifying predictive biomarkers to distinguish patients who would benefit from immunotherapy from those that may not respond to immunotherapy. Tumor immunogenicity factors such as DNA damage repair [28], MSI [29], TMB [16], neo-antigens [30], and human leukocyte antigen-presented tumor neo-antigens [31] have emerged as predictive biomarkers for immunotherapy. Moreover, tumor immune microenvironment is another important biomarker for immunotherapy. Therefore, we explored the relationship between immune checkpoint proteins and existing immunotherapeutic biomarkers to understand the role of ICGs in EC pathology and immunotherapy.

In this study, we first analyzed the relationship between 47 ICGs and TMB. The results showed positive correlation between TMB and seven prognostic ICGs. Further analysis showed positive correlation between MMR gene mutations and prognostic ICGs. Furthermore, we observed positive correlation between expression levels of *TNFRSF18*, *TNFRSF14*, and *BTLA* in EC patient tissues, TMB and neo-antigens. We also observed positive correlation between expression levels of adaptive immunity pathway genes (*CD8A*, *CD68*, *GZMB*, and *NOS2*) and most prognostic ICGs. These results demonstrate for the first time relationship between ICGs and immunotherapy biomarkers in EC tissues.

We then investigated the relationship between prognostic ICGs and clinical characteristics of EC patients. In general, prognostic ICGs were downregulated in advanced-stage EC tissues compared to early-stage EC tissues. Prognostic ICGs such as *PD-1, PD-L1,* and *IDO1* did not individually show significant association with OS. However, integrated analysis of multiple ICGs showed their association with prognosis. We also included *TNFRSF14* in the combination because of its correlation with prognosis and multiple biomarkers. EC patients belonging to the *PD-L1*^low^ *IDO1*^low^ *CTLA4* ^low^ *TNFRSF14*^high^ expression group showed the best prognosis, whereas, patients in the *PD-L1*^low^ *IDO1*^low^ *CTLA4*^low^ *TNFRSF14*^low^ expression group showed the worst prognosis. These results confirmed that the combination of PD-l1, IDO1, CTLA4, and *TNFRSF14* was a predictive biomarker signature to predict prognosis of EC patients.

Further analysis showed that *TNFRSF14* expression was significantly associated with tumor stage, mutant types, and histological types of EC. Therefore, *TNFRSF14* is a potential prognostic biomarker for endometrial cancer subtypes. *In vitro* experiments confirmed that *TNFRSF14* silencing in EC cells increased their proliferation, migration, invasiveness, and chemoresistance to 5-fluorouracil.

There are several limitations in our study. Firstly, this study was mainly based on analysis of public databases. It lacked clinical information regarding risk factors such as delayed menopause, early menarche, and chronic inflammation. Hence, the effects of these factors on the tumor immune microenvironment, expression of ICGs, and immunotherapeutic response could not be analyzed. Secondly, EC samples analyzed in this study were all from retrospective studies. Therefore, results of our study need to be confirmed by future prospective studies.

In summary, our study demonstrated significant association between prognostic ICGs, clinicopathological characteristics, and immunotherapeutic response factors in EC patients. Our results demonstrate that prognostic ICGs are potential predictive biomarkers to identify EC patients that may respond favorably to immunotherapy.

## MATERIALS AND METHODS

### Data download and preprocessing

Supplementary Table 1 shows RNA-seq data of 47 immune checkpoint genes in EC patient tissues (*n* = 543) from the TCGA database. The latest clinical follow-up information for the 543 EC patients (Supplementary Table 2) was downloaded on 2019.7.23 using the TCGA Genomic Data Commons application programming interface. The GSE77688 expression data (Supplementary Table 3) was downloaded from National Center for Biotechnology Information (NCBI). The TCGA RNA-seq data was pre-processed and (1) samples without clinical information or overall survival (OS) <30 days; (2) normal tissue samples; and (3) genes with 0 fragments per kilobase million (FPKM) in more than half of the samples were removed. After pre-processing, we included 521 EC samples from the TCGA dataset for further analysis. The GSE77688 data was pre-processed by removing normal tissue sample data. Moreover, Gene Expression Omnibus (GEO) platform information was used to map the chip probes to the human gene symbols.

### Analysis of the relationship between ICGs and prognosis of EC patients

We classified EC patients in the TCGA cohort into different groups based on expression levels of various ICGs. Then, we performed univariate Cox regression analysis to identify prognostic ICGs. Log rank test (*p* < 0.05) was used to compare differences in overall survival rates of EC patients with low and high expression levels of different ICGs. We also analyzed expression levels of ICGs in the GEO-EC dataset and identified prognostic ICGs using the same method.

### Analysis of the relationship between ICGs, TMB, and neoantigens

Spearman's Rank correlation method was used to evaluate the relationship between TMB and various ICGs. We also evaluated somatic mutation data from the TCGA database to determine the relationship between expression levels of neoantigens and ICGs in EC.

### Analysis of the relationship between prognosis and ICG-defined subtypes

We identified high expression (H) and low expression (L) groups according to the horizontal density distribution of the ICGs. Then, we classified ICGs into the high and low expression groups based on the deviation from the first main distribution interval (density peak) as the threshold. The H and L groups of *IDO1*, *CD274*, *CTLA4*, and *CD8A* were then integrated. Subsequently, patient samples were classified into four groups based on the three-gene classification and compared their survival rates.

### TNFRSF14 silencing by lentiviral-delivered RNA interference

Construction of hairpin-pLKO.1 vector (carrying a puromycin antibiotic resistance gene) containing short hairpin RNA (shRNA) sequences and production of shRNA viruses. The shRNAs targeting the TNFRSF14 coding sequences are as follows: shRNA #1 CCCTGTGACGATAAGAACGAT; and shRNA#2: GCACTCAGTAGCTTATCAAGCT. The control shRNA coding sequences are as follows: RFP, 59- CTACAAGACCGACATCAAGCT-39 and LacZ, 59-CCGTCA- TAGCGATAACGAGTT-39. for Lentiviral infections, adherent cells were treated with 0.5 mL of the virus followed by overnight incubation (37uC, 5% CO2) without removing the virus. The next day, viral medium was replaced with fresh medium containing puromycin (1 mg/mL) to select a population of resistant cells.

### Flow cytometry

The EC cells were trypsinized, washed with phosphate-buffered saline (PBS), and stained with the Annexin V-FITC Apoptosis Detection Kit (Beyotime, Shanghai, China) according to manufacturer’s instructions. The cells were dual-stained with propidium iodide and Annexin V-FITC in the dark for 30 mins. The stained cells were analyzed using the BDTM LSRII flow cytometer (BD Biosciences, San Jose, CA, USA). The percentage of apoptotic cells were analyzed using the Cell Quest software (BD Biosciences, San Jose, CA, USA).

### Wound healing and transwell assays

For the wound healing assay, EC cells were seeded in six-well plates. When the cultures reached 95% confluence, the cell monolayer was gently scratched with a sterile 200-μm plastic pipette tip, and the wound was photographed at 0 h. The plates were incubated for a further 24 h and the wound was photographed again.

For the Transwell migration and invasion assays, 4 × 10^4^ cells in DMEM medium without serum were seeded in the upper chamber of Transwell with membranes coated without/with Matrigel (BD Biosciences, San Jose, CA, USA). We added 600 μL of DMEM medium with 10% fetal bovine serum into the lower chamber. The Transwell chambers were incubated for 24 h. Then, after removing the cells in the upper chamber, the cells on the the underside of the membrane or in the matrigel were fixed for 30 mins, stained with 0.1% crystal violet, and counted under a light microscope.

### Western blotting

The EC cells were washed twice with cold phosphate-buffered saline and total proteins were extracted by incubation in NP-40 lysis buffer for 30 min at 4°C. The protein concentration was measured using bicinchoninic acid (BCA) assay kit (Thermo). Equal amounts of protein lysates were separated by electrophoresis in a premade 8–12% sodium dodecyl sulfate-polyacrylamide mini gel (SDS-PAGE) and the separated proteins were transferred onto a polyvinylidene difluoride (PVDF) membrane. The membranes were blocked with 5% skimmed milk and then incubated with primary antibodies overnight at 4^o^C. We then incubated membranes with HRP-conjugated secondary antibodies at room temperature for 1 h. Then, the blots were developed using ECL chemiluminescence kit. The protein bands were imaged and quantified.

### Statistical analysis

Shapiro-Wilk normality test was used to determine normality of the variables. The normally distributed variables between groups were analyzed using unpaired Student’s *t*-test, and the non-normally distributed variables were analyzed using Mann-Whitney *U* test. The data for multiple groups was compared using Kruskal-Wallis test and one-way ANOVA for non-parametric and parametric methods, respectively. Spearman correlation analysis was performed to determine the association between ICGs and other clinical variables. Two-sided Fisher’s exact test was used to analyze the contingency table. Benjamini-Hochberg method was used to convert *P*-values into false discovery rate (FDR). Kaplan-Meier survival curves and log rank test were used to compare OS rates of different EC patient subgroups. *P* < 0.05 was considered statistically significant. All statistical analyses were performed using the R software version 3.4.3 with default parameters, unless otherwise specified.

### Availability of data and material

The data used to support the findings of this study are available from the corresponding author on reasonable request.

## Supplementary Materials

Supplementary Figure 1

Supplementary Table 1

Supplementary Table 2

Supplementary Table 3

Supplementary Table 4
