# Supplementary material for: Identification of an immune checkpoint gene signature that accurately predicts prognosis and immunotherapy response in endometrial carcinoma
Source: Aging (Albany NY). 2021 Jun 22;13(12):16696–712. doi: 10.18632/aging.203189 (PMC8266314; doi:10.18632/aging.203189)
Supplement: Supplementary Figure 1 [file aging-13-203189-s001.pdf]

SUPPLEMENTARY FIGURE

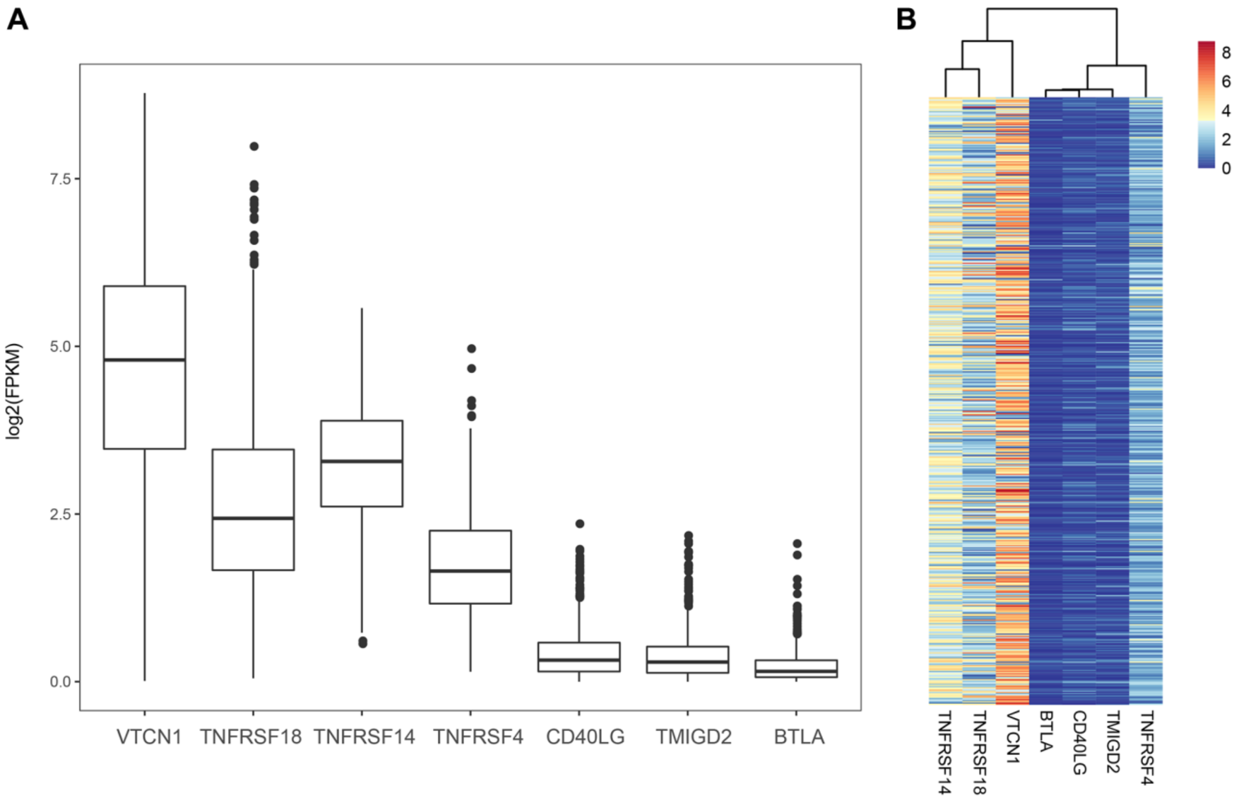

Supplementary Figure 1. Heat map of 7 ICGs divided into medium expression level group and low expression group.
