# Supplementary material for: Identification of an immune checkpoint gene signature that accurately predicts prognosis and immunotherapy response in endometrial carcinoma
Source: Aging (Albany NY). 2021 Jun 22;13(12):16696–712. doi: 10.18632/aging.203189 (PMC8266314; doi:10.18632/aging.203189)
Supplement: Supplementary Table 4 [file aging-13-203189-s005.pdf]

Supplementary Table 4. The relationship between adaptive immune resistance pathway genes and ICGs in EC tissues.

	CD8A_cor	CD68_cor	GZMB_cor	NOS2_cor	CD8A_corP	CD68_corP	GZMB_corP	NOS2_corP
TNFRSF18	0.024508195	0.092705761	0.049385124	0.036973425	0.576741858	0.034385362	0.260497685	0.399678767
TNFRSF4	0.431698527	0.412209402	0.380313547	0.069620288	4.59E-25	8.65E-23	2.25E-19	0.112462352
TNFRSF14	0.477396407	0.28266388	0.277174491	0.254448726	5.16E-31	5.00E-11	1.21E-10	3.83E-09
TNFRSF25	0.058416885	0.004305586	0.012506425	0.005350553	0.183082207	0.921899548	0.77580508	0.903028765
TNFRSF9	0.715021879	0.252897023	0.376841563	0.148380255	1.01E-82	4.80E-09	5.03E-19	0.000679965
TNFRSF8	0.209466295	0.170492682	0.219337331	0.004574085	1.41E-06	9.20E-05	4.28E-07	0.917046182
VTCN1	–0.102147737	0.000384812	–0.102757137	–0.032320474	0.019697105	0.993008712	0.018973313	0.461640069
CD160	0.062842968	–0.005019849	0.00867619	0.041455219	0.152032762	0.908995461	0.843384817	0.344982538
CD48	0.756262111	0.606017374	0.563062773	0.100379093	1.10E-97	1.51E-53	6.57E-45	0.021935527
CD244	0.854266361	0.4428357	0.537919957	0.282371149	1.40E-149	1.97E-26	2.06E-40	5.25E-11
TNFSF18	0.098955769	0.036571254	0.054753608	0.132550314	0.023894037	0.404828884	0.212137773	0.002432312
TNFSF4	0.071861062	0.059676481	0.042230498	0.002304044	0.101330296	0.173804084	0.336028	0.958158558
CD28	0.524601285	0.250444121	0.277200437	0.046694165	3.53E-38	6.82E-09	1.21E-10	0.287403669
CTLA4	0.547898643	0.302546207	0.479223484	0.104228422	3.76E-42	1.72E-12	2.86E-31	0.017320686
ICOS	0.775104678	0.307636796	0.413363095	0.184488464	1.47E-105	6.98E-13	6.41E-23	2.26E-05
PDCD1	0.861997544	0.524208583	0.685019708	0.199897632	2.94E-155	4.09E-38	2.07E-73	4.26E-06
HHLA2	–0.011471527	–0.010853193	–0.045943781	0.046134656	0.793921195	0.804799112	0.295228617	0.293224818
CD200	0.066168249	–0.075420432	–0.064593542	–0.028908832	0.131466481	0.085465408	0.140920542	0.510275298
BTLA	0.530456486	0.250357202	0.327785489	0.134816962	3.79E-39	6.90E-09	1.63E-14	0.002042694
CD200R1	0.596510714	0.147858117	0.23365301	0.120666348	1.60E-51	0.000710633	6.84E-08	0.005820458
TIGIT	0.912122396	0.487411317	0.588115976	0.265559242	3.75E-203	1.93E-32	8.63E-50	7.37E-10
CD80	0.420846473	0.279833315	0.214913803	0.02667123	8.85E-24	7.91E-11	7.36E-07	0.54356692
CD86	0.621854364	0.478325584	0.439589343	0.048657956	4.46E-57	3.82E-31	4.99E-26	0.267590855
HAVCR2	0.837876527	0.568336964	0.585946326	0.159557516	1.46E-138	6.67E-46	2.38E-49	0.00025551
IDO1	0.401433964	0.10267899	0.197063979	0.417707913	1.36E-21	0.019064813	5.85E-06	2.04E-23
IDO2	0.031888295	0.000308607	0.015747821	–0.013826013	0.467654675	0.994393176	0.719887175	0.752881871
CD274	0.538039915	0.257186538	0.311429187	0.339618569	1.97E-40	2.57E-09	3.51E-13	1.57E-15
TNFSF15	0.066137578	–0.037809495	–0.033604282	0.10797318	0.131645816	0.389098777	0.444029901	0.013669747
NRP1	0.091210188	0.009409516	–0.024042618	–0.037718406	0.037410972	0.830340347	0.584005056	0.390243141
CD44	0.105868843	0.138785654	0.069620283	0.050457972	0.015627773	0.001495199	0.112462379	0.250271259
CD27	0.759005964	0.649177197	0.669807531	0.111594942	8.73E-99	1.15E-63	4.16E-69	0.010801674
LAG3	0.841066245	0.698516245	0.726408154	0.188674205	1.32E-140	1.87E-77	1.41E-86	1.46E-05
CD276	–0.139356511	–0.107366503	–0.116958595	0.00891636	0.001428625	0.014210775	0.007531199	0.839107757
LGALS9	0.227468476	0.105572178	0.11531322	0.168648756	1.53E-07	0.015922725	0.008425017	0.000109744
TMIGD2	0.365543851	0.181129218	0.224595226	0.082785597	6.47E-18	3.20E-05	2.21E-07	0.058982987
TNFSF9	0.292541259	0.179501018	0.24503206	0.205611834	9.70E-12	3.77E-05	1.46E-08	2.22E-06

CD70	0.484833006	0.28758628	0.386339774	0.101225577	4.54E-32	2.23E-11	5.43E-20	0.020838067
TNFSF14	0.108659044	–0.034457418	0.002660625	0.028741907	0.01308002	0.432542008	0.951690417	0.512721633
LAIR1	0.603226219	0.474489502	0.489124671	0.040113275	6.03E-53	1.31E-30	1.09E-32	0.360837096
KIR3DL1	0.059540573	0.023940614	0.063261107	0.025357677	0.174787987	0.585602014	0.149319881	0.563600115
CD40	0.333467892	0.273046228	0.234830173	0.194138373	5.36E-15	2.32E-10	5.85E-08	8.08E-06
ICOSLG	0.020172574	0.068887923	0.023330296	0.005648767	0.645955303	0.116302215	0.595199008	0.897652934
ADORA2A	0.177341149	0.082878158	0.132915209	0.021886256	4.69E-05	0.05869931	0.002365327	0.618186
CD40LG	0.386902313	0.086009635	0.140727986	0.053342867	4.75E-20	0.049747555	0.001279666	0.224169868
